# Ca^2+^-permeable mechanosensitive channels MCA1 and MCA2 mediate cold-induced cytosolic Ca^2+^ increase and cold tolerance in Arabidopsis

**DOI:** 10.1038/s41598-017-17483-y

**Published:** 2018-01-11

**Authors:** Kendo Mori, Na Renhu, Maho Naito, Aki Nakamura, Hayato Shiba, Tsuyoshi Yamamoto, Takuya Suzaki, Hidetoshi Iida, Kenji Miura

**Affiliations:** 1 Department of Biology, Tokyo Gakugei University, 4-1-1 Nukui kita-machi, Koganei, Tokyo, 184-8501 Japan; 2 Graduate School of Life and Environmental Sciences, University of Tsukuba, 1-1-1 Tennoudai, Tsukuba, 305-8572 Japan

## Abstract

Cold shock triggers an immediate rise in the cytosolic free calcium concentration ([Ca^2+^]_cyt_) in *Arabidopsis thaliana* and this cold-induced elevation of [Ca^2+^]_cyt_ is inhibited by lanthanum or EGTA. It is suggested that intracellular calcium mainly contributes to the cold-induced [Ca^2+^]_cyt_ response by entering into the cytosol. Two calcium-permeable mechanosensitive channels, MCA1 and MCA2 (*mid1*-complementing activity), have been identified in Arabidopsis. Here, we demonstrate that MCA1 and MCA2 are involved in a cold-induced increase in [Ca^2+^]_cyt_. The cold-induced [Ca^2+^]_cyt_ increase in *mca1* and *mca2* mutants was markedly lower than that in wild types. The *mca1 mca2* double mutant exhibited chilling and freezing sensitivity, compared to wild-type plants. Expression of *At5g61820*, *At3g51660*, and *At4g15490*, which are not regulated by the *CBF/DREB1s* transcription factor, was down-regulated in *mca1 mca2*. These results suggest that MCA1 and MCA2 are involved in the cold-induced elevation of [Ca^2+^]_cyt_, cold tolerance, and *CBF/DREB1*-independent cold signaling.

## Introduction

Calcium ions are used as secondary messengers in eukaryotic cells. The cytosolic Ca^2+^ concentration, [Ca^2+^]_cyt_, fluctuates in response to a variety of stimuli, including mechanical stimulation, hormones, pathogens, light, and abiotic stresses such as low temperature^1–3^. The stimulus-specific spatiotemporal patterning of [Ca^2+^]_cyt_ dynamics is called the Ca^2+^ signature^4^, and to create these signatures, Ca^2+^ influx channels and Ca^2+^ efflux transporters that permit transient increases in [Ca^2+^]_cyt_ are required^5^.

How plant cells generate stimulus-specific Ca^2+^ signals remains unknown. To identify the spatiotemporal patterning of [Ca^2+^]_cyt_ dynamics, recombinant aequorin has been introduced as a reporter of [Ca^2+^]_cyt_ changes in plant systems^6^. In Arabidopsis plants expressing aequorin in the cytoplasm, low temperature triggers an immediate and transient rise in [Ca^2+^]_cyt_^6–8^. The final temperature and cooling rate are important for sensing low temperature in Arabidopsis^9^. In mammals, many TRP (transient receptor potential) channels, which are a specific class of ion channels, function as intracellular Ca^2+^ release channels^10^. Some of these channels also function as thermosensors^10^, and TRPA1 seems to act as a sensor for cold^11–13^. Although no proteins with high similarity to TRP channels have been identified in land-plant genomes, the genes for Cr-TRP proteins are encoded in the genomic sequence of the alga *Chlamydomonas reinhardtii* and show functional properties that are similar to those of mammalian TRP channels^14^.

Two Ca^2+^-permeable mechanosensitive channels, named MCA1 and MCA2 (*mid1*-complementing activity 1 and 2), have been identified in Arabidopsis^15–19^. Both *MCA1* and *MCA2* complement deficiency of Ca^2+^ uptake in yeast cells lacking a Ca^2+^ channel composed of the Mid1 and Cch1 subunits^15,16^. It should be noted that this complementation activity is detected under conditions that allow the Mid1/Cch1 channel to function as the sole Ca^2+^ influx system in yeast cells, suggesting that MCA1 and MCA2 can directly mediate Ca^2+^ influx in the cells lacking both Mid1 and Cch1. Electrophysiological studies have shown that both MCA1 and MCA2 produce stretch-activated currents when expressed in *Xenopus laevis* oocytes^17^. These results with yeast cells and *Xenopus* oocytes suggest that MCA1 and MCA2 mediate Ca^2+^ influx as mechanosensitive channels, and are not accessory factors that facilitate Ca^2+^ influx. Overexpression of *MCA1* enhances an increase in [Ca^2+^]_cyt_ upon hypoosmotic shock^15^. The *mca2* mutant exhibits a defect in Ca^2+^ uptake from the roots^16^. Structurally, MCA1 and MCA2 have 74% identity and 89% similarity in amino acid sequences^15^. Both have a single transmembrane segment and an EF-hand-like motif and coiled-coil motif in the N-terminal region, as well as a plac8 motif in the C-terminal region^15,18^. MCA1-GFP and MCA2-GFP are localized to the plasma membrane^15^. MCA1 and MCA2 form a homotetramer^19,20^. Topological analysis has indicated that the EF-hand-like motif, the coiled-coil motif, and the plac8 motif are present in the cytoplasm^18^, suggesting that both channels recognize intracellular Ca^2+^. The *MCA* genes are conserved in the plant kingdom^21^, and an increase in [Ca^2+^]_cyt_ as a result of hypo-osmotic shock is mediated by MCA proteins in rice and tobacco^22,23^.

Application of the patch-clamp technique has demonstrated that Ca^2+^-permeable channels are transiently activated by cold shock in Arabidopsis mesophyll cells^7^. In plants, extracellular freezing causes dehydration and mechanical stresses on the plasma membrane, and cold-acclimated plant plasma membranes become resistant to mechanical stress^24^. Expression of *CBF2* is induced not only by cold, but also by mechanical stress^25^. Therefore, it is assumed that mechanical stress may be one of the factors involved in cold acclimation.

Three CBF/DREB1 (C-repeat binding factor/DRE binding factor 1) transcription factors have been extensively studied. They belong to the AP2/ERF (Apetala/ethylene-responsive factor) superfamily and are important factors for cold acclimation in plants^26^. *CBF/DREB1* genes are rapidly and transiently induced after cold treatment^27^, and overexpression of *CBF/DREB1* constitutively enhances freezing tolerance^28,29^. Under cold stress, CBF/DREB1 proteins bind to CRT/DRE *cis*-elements in the promoter of cold-regulated (*COR*) genes and induce transcription^28^. However, gene expression analyses reveals that only 6.5% of the total *COR* genes are regulated by *CBF/DREB1*^30^. In addition to *CBF/DREB1* genes, 27 transcription factors that were up-regulated at an early stage after cold treatment were considered as first-wave transcription factors^30^. Use of the *cbf1/2/3* triple mutant showed that six first-wave transcription factors are partially regulated by *CBF/DREB1*, whereas the transcription factors HSFC1, ZAT12, and CZF1, which regulate cold-regulated genes^30,31^, are not regulated by *CBF/DREB1*^32^. As acclimated *cbf1/2/3* triple mutants are more tolerant of freezing stress than non-acclimated ones^33^, and the expression of a large number of cold-regulated genes is not affected by the *cbf1/2/3* triple mutation^32^, a *CBF/DREB1-*independent pathway may control cold tolerance. Overexpression of *HSFC1* enhances cold tolerance without an increase in expression of *CBF1*, *CBF2*, or *CBF3*^30^, suggesting that *HSFC1* is one of the important transcription factors controlling non-*CBF/DREB1* regulons and cold tolerance.

Here, we demonstrate that MCA1 and MCA2 are involved in a transient rise in [Ca^2+^]_cyt_ upon cold shock. The cold-induced increase in [Ca^2+^]_cyt_ was smaller in the *mca1* and *mca2* mutants than in the Col-0 wild type. The *mca1 mca2* double mutant exhibited increased sensitivity to chilling and freezing stresses. These results suggest that MCA1 and MCA2 are involved in cold-induced Ca^2+^ influx and that the reduced [Ca^2+^]_cyt_ increase caused by the *mca1* and *mca2* mutations affects cold acclimation. As the *CBF/DREB1* genes and their regulon genes were not down-regulated in the *mca1 mca2* mutant, MCA may not be involved in the regulation of *CBF/DREB1-*dependent cold signaling.

## Results

### MCA1 and MCA2 are involved in a cold-induced [Ca^2+^]_cyt_ increase

**Figure 1:**
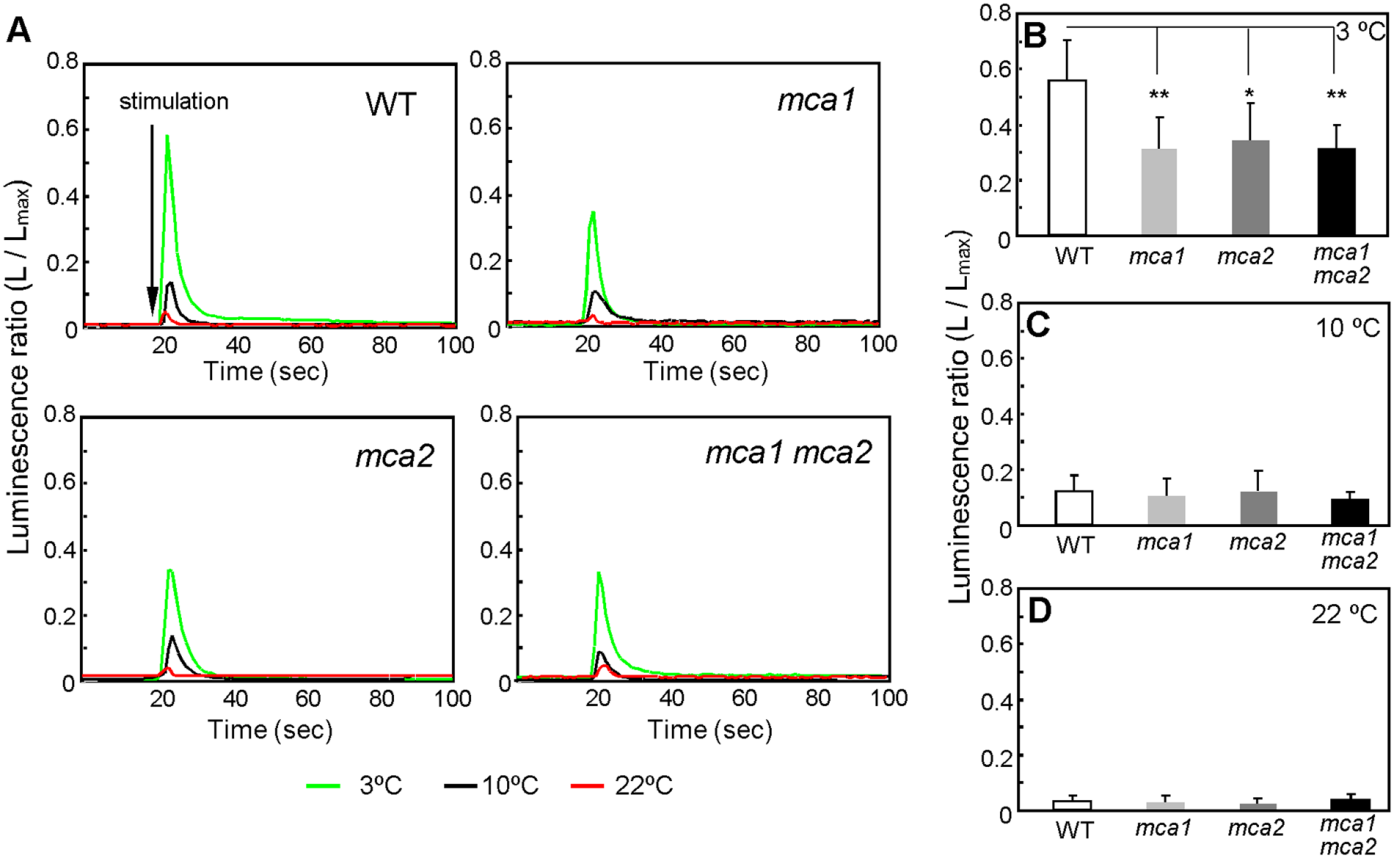
Transient cold-induced increase in cytosolic Ca^2+^ is lower as a result of the *mca* mutation. (**A**) Relative luminescence of plants harboring aequorin was measured before and after the addition (indicated by the vertical arrow) of precooled solution (3 °C or 10 °C) or room temperature solution (22 °C). The figures are of representative data. The peak luminescence after the addition of solution at 3 °C (**B**, *n* ≥ 10), 10 °C (**C**, *n* ≥ 9), and 22 °C (**D**, *n* ≥ 17) is shown. Data represent the means ± SD. *n* indicates the number of seedlings. *p < 0.05; **p < 0.005 versus the wild type. Significance was determined using unpaired Student’s *t* tests.

To monitor changes in [Ca^2+^]_cyt_ (the cytosolic concentration of Ca^2+^), Arabidopsis seedlings expressing aequorin, a Ca^2+^ indicator^15^, that had been immersed in MS medium (400 µl) at 22 °C were exposed to low temperatures by the addition of MS medium (500 µl) kept at 3, 10, or 22 °C. [Ca^2+^]_cyt_ in the wild type was significantly increased by a 3 °C shock (Fig. 1A green line and B), moderately by a 10 °C shock (Fig. 1A black line and C), and just a little by a 22 °C shock (Fig. 1A red line and D). On the other hand, the magnitude of the cold-induced [Ca^2+^]_cyt_ increase was markedly lower in the *mca1*, *mca2*, and *mca1 mca2* mutants (Fig. 1). Small increases observed in response to the 22 °C shock in both the wild type and the mutants could be a consequence of mechanical stress rather than cold stress, because MS medium (a fluid) was added to induce the response. These results suggest that MCA1 and MCA2 contribute to a [Ca^2+^]_cyt_ increase upon cold shock.

**Figure 2:**
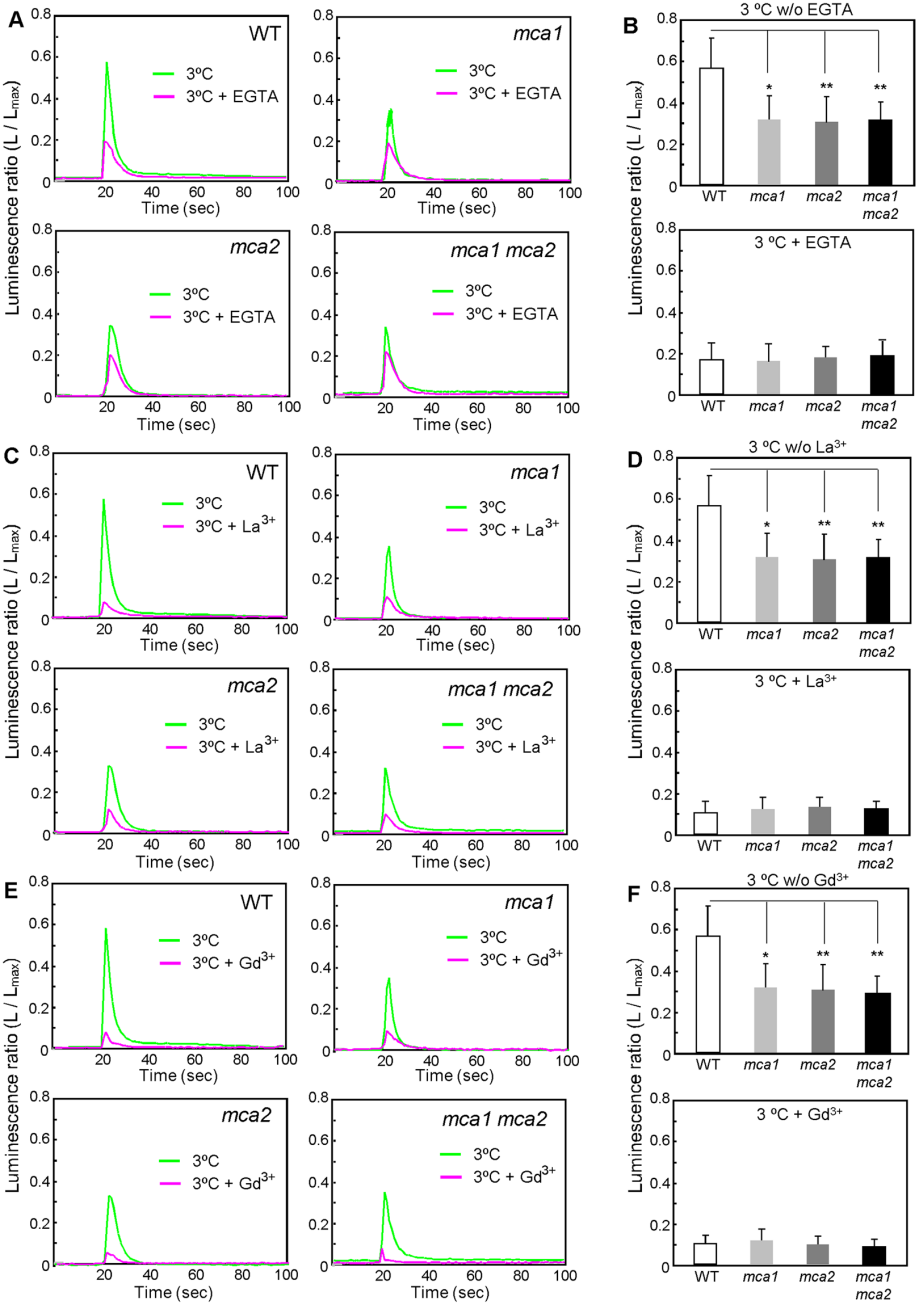
Effect of channel blockers and a Ca^2+^ chelator on the cold-shock-induced [Ca^2+^]_cyt_ increase. Thirty minutes before cold shock, 5 mM EGTA (**A**), 1 mM La^3+^ (**C**), or 1 mM Gd^3+^ (**D**) was added to the medium. Then, the relative luminescence of a plant harboring aequorin was measured, as in Fig. 1, before and after the application of precooled solution (3 °C). The peak luminescence after the addition of the solution at 3 °C with or without 5 mM EGTA (**B**, *n* ≥ 17), 1 mM La^3+^ (**D**, *n* ≥ 9), or 1 mM Gd^3+^ (**F**, *n* ≥ 9) is shown. Data represent the means ± SD. *p < 0.05; **p < 0.005 versus the wild type in each treatment. Significance was determined using unpaired Student’s *t* tests.

Since MCA1 and MCA2 are present in the plasma membrane^15,16^, the cold-induced [Ca^2+^]_cyt_ increase could be brought about by Ca^2+^ influx. To examine this possibility, we preincubated seedlings for 30 min in MS medium including either a Ca^2+^ chelator, EGTA, or a plasma membrane ion channel blocker, La^3+^ or Gd^3+^, and then monitored changes in [Ca^2+^]_cyt_ upon cold shock. As expected, the cold-induced [Ca^2+^]_cyt_ increase was inhibited by EGTA (Fig. 2A,B), La^3+^ (Fig. 2C,D), and Gd^3+^ (Fig. 2E,F) in the wild type and in all the *mca* mutants, although the inhibition rates of the wild type were greater than those of the *mca* mutants. It should also be noted that significant [Ca^2+^]_cyt_ increases remained in all the *mca* mutants, as well as in the wild type, suggesting that there is another cold-induced Ca^2+^ transport system(s) that is insensitive to the blockers we used in the plasma membrane, or that is in the intracellular compartment.

**Figure 3:**
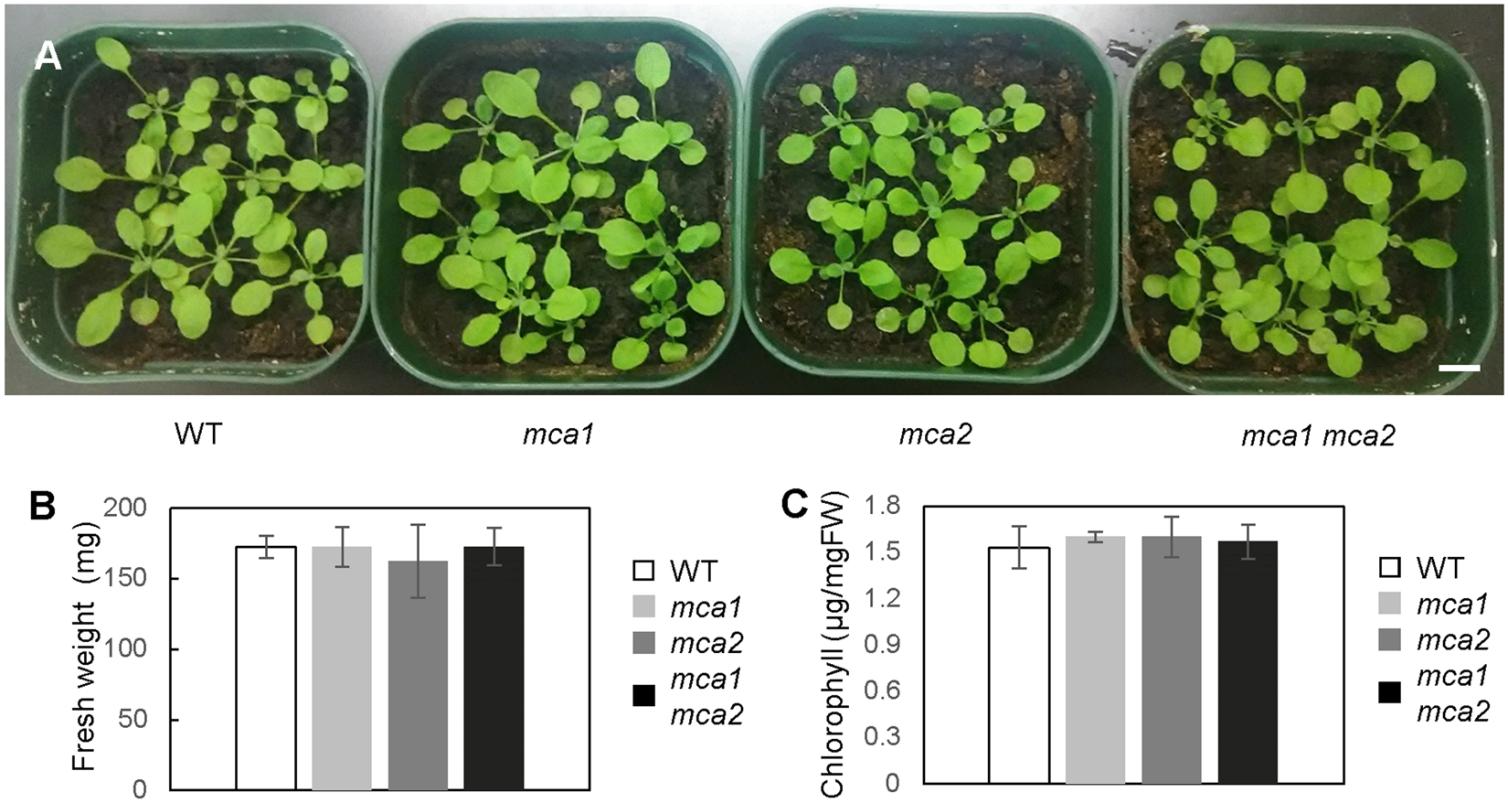
The growth of the *mca1 mca2* mutant is normal at room temperature. (**A**) Representative 3-week old plants of wild type, *mca1*, *mca2*, and *mca1 mca2* mutants (nine plants in each pot) are displayed. The bar indicates 1-cm length. Fresh weight (**B**) and chlorophyll content (**C**) of each plant were measured. Values represent the means ± SD (*n* = 10). No significant difference was observed with an unpaired Student’s *t-*test.

Even though the *mca* mutants exhibited a reduced cold-induced [Ca^2+^]_cyt_ increase, the mutants looked healthy when they grew under normal conditions (Fig. 3A). To examine whether the *mca* mutation affects plant growth under normal conditions, fresh weight and chlorophyll contents were measured (Fig. 3B,C). The *mca1*, *mca2*, and *mca1 mca2* plants had similar values, as did the wild type, suggesting that plant development in the aerial part is unaffected by *MCA1* and *MCA2*.

### Mutations in *MCA1* and *MCA2* result in cold sensitivity

**Figure 4:**
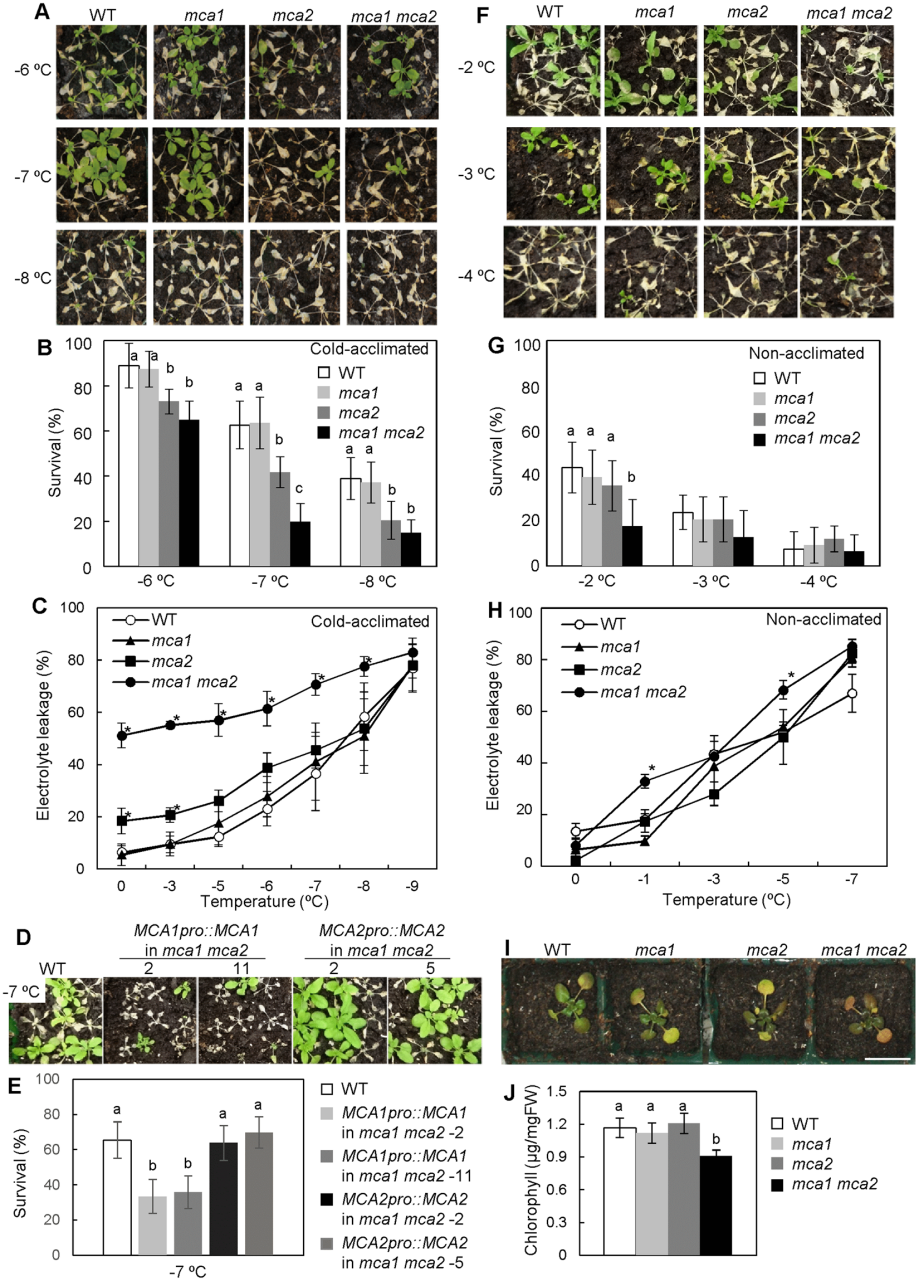
The *mca1 mca2* double mutant exhibited sensitivity to cold stress. (**A**) Freezing sensitivity of the *mca1 mca2* double mutant after cold acclimation. Three-week-old plants were incubated at 4 °C for 1 week, then plants were used for freezing treatment. Photographs are representative plants 7 days after 4-h exposure to the indicated temperature. (**B**) Survival rates were determined for 9 plants after freezing treatment at the indicated temperature. The survival ratio was calculated from 9 plants per pots. Data represent the means ± SD calculated from the data of 9 independent experiments. Differences between the values of each treatment were evaluated by one-way ANOVA followed by the Tukey-Kramer test. Difference of alphabet letters at each temperature indicates statistically significant difference (*p* < 0.05). (**C**) Electrolyte leakage from cold-acclimated wild-type, *mca1*, *mca2*, and *mca1 mca2* plants after exposure to the indicated temperature (programmed to cool at a rate of 2 °C h^−1^). Data represent the means ± SD (*n* = 4 leaves, each from a different plant). *p < 0.05 compared with the value of wild type at each temperature (one-way ANOVA followed by the Tukey-Kramer test). Data were representative experiments from 3 biological independent experiments. (**D**) Freezing sensitivity of the *mca1 mca2* double mutant harboring *MCA1pro::MCA1* (lines #2 and #11) or *MCA2pro::MCA2* (lines #2 and #5). Photographs are representative plants 7 days after 4-h exposure to −7 °C. (**E**) The survival ratio at −7 °C was calculated from 9 plants per pots. Data represent the means ± SD calculated from the data of 9 independent experiments. Difference between values of each treatment were evaluated by one-way ANOVA followed by the Tukey-Kramer test. Difference of alphabet letters at each temperature indicates statistically significant difference (*p* < 0.05). (**F**) Freezing sensitivity of the *mca1 mca2* double mutant without cold acclimation. Three-week-old plants were treated with a freezing temperature. Photographs are representative plants after freezing treatment. Photographs are representative plants 7 days after 1-h exposure to the indicated temperature. (**G**) The survival ratio was calculated from 9 plants per pots. Difference between values of each treatment were evaluated by one-way ANOVA followed by the Tukey-Kramer test. Difference of alphabet letters at each temperature indicates statistically significant difference (*p* < 0.05). (**H**) Electrolyte leakage from non-acclimated wild-type, *mca1*, *mca2*, and *mca1 mca2* plants after exposure to the indicated temperature. Data represent the means ± SD (*n* = 3 leaves, each from a different plant). *p < 0.05 compared with the value of wild types at each temperature (ANOVA followed by the Tukey-Kramer test). (**I**) Photographs are of representative wild type, *mca1*, *mca2*, and *mca1 mca2* mutants after incubation at 4 °C for 1 month. Five-day-old plants were incubated at 4 °C for 1 month. (**J**) The chlorophyll content of the plants was determined. Values represent the means ± SD (*n* = 7 plants per each genotype). Difference of alphabet letters indicates a significant difference (*p* < 0.05) as determined by one-way ANOVA followed by the Tukey-Kramer test.

Since the *mca* mutants exhibited a reduced cold-induced [Ca^2+^]_cyt_ increase, we investigated their cold sensitivity. Three-week-old plants were incubated at 4 °C for 1 week to acclimate to cold stress. These plants were then exposed to freezing temperatures (Fig. 4A,B). Before this exposure, *mca1*, *mca2*, *mca1 mca2* and the wild-type plants looked healthy (Fig. 3A). After this exposure, the survival of the *mca1* mutant was similar to that of the wild type. On the other hand, the *mca2* mutant exhibited a freezing-sensitive phenotype (Fig. 4A,B). Furthermore, the *mca1 mca2* double mutant was more sensitive to freezing stresses than the *mca2* mutant (Fig. 4A,B). Electrolyte leakage from the *mca1 mca2* double mutant was much higher (approximately 50%) than that of the *mca* single mutants and the wild type, even before it was subjected to freezing temperatures, and it increased as the freezing temperature was lowered (Fig. 4C). At every freezing temperature we employed (−3 to −9 °C), the leakage was greatest in the double mutant. To confirm whether the freezing sensitivity of the *mca1 mca2* mutant was caused by the mutation in *MCA1* or *MCA2*, complement lines were produced. *MCA1pro::MCA1* or *MCA2pro::MCA2* was expressed in the *mca1 mca2* mutant (Fig. 4D,E). Because the own promoter was used for expression of *MCA1* or *MCA2*, the expression level of *MCA1* in the complement lines, *MCA1pro::MCA1* in *mca1 mca2*, was similar to that of wild type and the *mca2* mutant (Figure S1). The expression level of *MCA2* in the complement lines, *MCA2pro::MCA2* in *mca1 mca2*, was slightly higher than that of WT and *mca1* (Figure S1). The sensitivity of *MCA2pro::MCA2-*expressing *mca1 mca2* mutant was recovered (Fig. 4E). On the other hand, the survival ratio of *MCA1pro::MCA1-*expressing *mca1 mca2* was similar to that of *mca2* (Fig. 4E). The wild-type, *mca1*, *mca2*, *and mca1 mca2* plants without cold acclimation were also treated with a freezing temperature for 1 h (Fig. 4F). Before acclimation, the cold sensitivity of the *mca1 mca2* plants was slightly greater than that of wild-type, *mca1* and *mca2* plants (Fig. 4G). Furthermore, electrolyte leakage of the *mca1 mca2* mutant was a little higher than that of the wild type (Fig. 4H). These results suggest that MCA mainly functions in the regulation of cold tolerance during cold acclimation.

To examine whether the mutant exhibits chilling sensitivity, wild-type, *mca1*, *mca2*, and *mca1 mca2* plants were incubated at 4 °C for 1 month under continuous light conditions. The leaves of the *mca1 mca2* double mutant looked unhealthy (Fig. 4I). Thus, to quantify chilling sensitivity, chlorophyll content was measured. The chlorophyll content in the *mca1 mca2* double mutant was approximately three-fourths that of the wild type (Fig. 4J). No detectable difference was observed between the wild type and the *mca* single mutants. These results suggest that the double mutation in *MCA1* and *MCA2* results in hypersensitivity to cold stress in Arabidopsis plants.

### Down-regulation of cold-inducible genes is governed by a non-*CBF/DREB1* regulon

**Figure 5:**
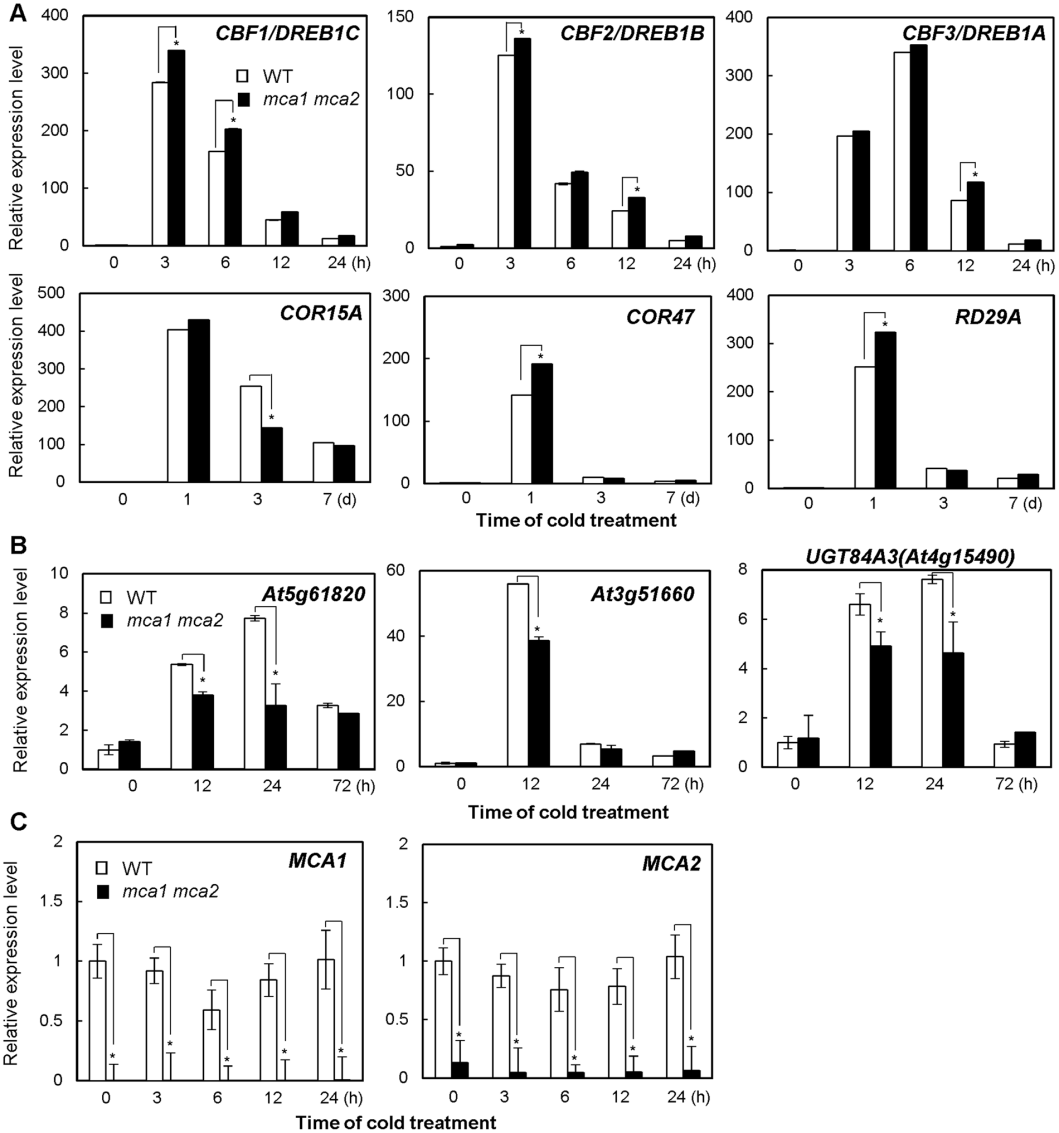
The expression levels of several cold-regulated genes, which are not regulated by *CBF/DREB1*, were reduced in the *mca1 mca2* double mutant. (**A**) Relative mRNA transcript levels of *CBF/DREB1* and its regulon genes, *COR15A*, *COR47*, and *RD29A*, in wild-type and *mca1 mca2* plants were determined by quantitative RT-PCR analyses. Three-week-old plants grown at 24 °C were incubated at 4 °C for the indicated time. Values represent the means ± SD (*n* *=* 3) from representative experiments from 3 biologically independent experiments. (**B**) Relative expression levels of cold-regulated genes that are not *CBF/DREB1*-regulon genes^30^ were determined by quantitative RT-PCR analyses. Values represent the means ± SD (*n* *=* 3) from representative experiments from 3 biologically independent experiments. (**C**) The expression levels of *MCA1* and *MCA2* in wild types and *mca1 mca2* double mutants were investigated by quantitative RT-PCR analyses. An asterisk indicates a significant difference from wild-type plants at each point (*p* < 0.05) as determined by unpaired Student’s *t*-tests.

The expression of the *CBF/DREB1* genes and their regulon genes, *COR15* *A*, *COR47*, and *RD29A*, was investigated. To perform this, three-week-old wild-type, *mca1*, *mca2*, and *mca1 mca2* plants were exposed to cold at 4 °C for appropriate periods, and RNA prepared from the plants was subjected to a quantitative RT-PCR analysis. Interestingly, the expression of these genes was slightly up-regulated in the *mca1 mca2* double mutant, especially soon after the start of the cold treatment (Fig. 5A). This increase could be the consequence of a compensatory response caused by a lack of the function of MCA1 and MCA2 and suggests that both proteins may control another cold signaling pathway. *CBF/DREB1* and its regulatory genes are only partly responsible for the acquisition of tolerance to freezing stress for cold acclimation^30^. Therefore, we examined the expression of cold-inducible genes that are governed by *HSFC1* but not by *CBF2*^30^, such as *At5g61820*, *At3g51660*, and *At4g15490*, which encode an unknown protein, a tautomerase/MIF superfamily protein, and a UDP-glycosyltransferase superfamily protein, respectively. Figure 5B shows that the expression of the three genes was significantly down-regulated in the *mca1 mca2* double mutant. Expression of MCA1 and MCA2 themselves was unchanged upon cold shock (Fig. 5C). According to these results, it is plausible that MCA1 and MCA2 mediate cold tolerance by participating in a pathway other than the *CBF/DREB1* pathway.

## Discussion

In the present study, we have demonstrated that MCA1 and MCA2 play a role in the transient rise in [Ca^2+^]_cyt_ upon cold shock and are also involved in chilling and freezing tolerance. The *mca1 mca2* double mutant exhibited a lower cold-induced increase in [Ca^2+^]_cyt_ than the wild type (Fig. 1), as well as an increased sensitivity to cold stress (Fig. 4). Although the *mca1* and *mca2* single mutants exhibited a lower cold-induced increase in [Ca^2+^]_cyt_ like the double mutant, the *mca1* single mutant did not show a cold sensitive phenotype similar to that of the *mca2* single and *mca1 mca2* double mutants. We speculate that a reason for this discrepancy may be a difference in the spatial expression patterns of *MCA1* and *MCA2* in Arabidopsis plants^16^, as explained in more detail in a later paragraph. As for the regulation of gene expression, *CBF/DREB1* genes and their regulon genes were not down-regulated in the *mca1 mca2* double mutant (Fig. 5), suggesting that MCA may not regulate *CBF/DREB1*-dependent cold signaling.

Different stimuli produce different patterns of Ca^2+^ elevation and oscillations with different frequencies, and these are called Ca^2+^ signatures. As shown in Fig. 1, low-temperature stress stimulates a transient increase in [Ca^2+^]_cyt_^34^. MCA1 and MCA2 have been identified as plasma membrane proteins involved in Ca^2+^ influx in response to mechanical stimuli, such as touch, gravity, flexure, and turgor^15,16^. In the *mca1* or *mca2* mutant, the magnitude of the cold-induced [Ca^2+^]_cyt_ increase was lower, by approximately 40%, than that in the wild type (Fig. 1). These results suggest that MCA1 and MCA2 are partially involved in Ca^2+^ influx in response to cold shock. Application of a mechanosensitive Ca^2+^ channel blocker, Gd^3+^, prevents the induction of cold-regulated genes^35^. As two Ca^2+^ channel inhibitors, La^3+^ and Gd^3+^, still reduced the cold-induced [Ca^2+^]_cyt_ increase in the *mca* mutants, and the *mca* mutations were unable to block [Ca^2+^]_cyt_ increases completely (Fig. 2), other cold-activatable Ca^2+^ transport system(s) must exist in the plasma membrane and/or organellar membranes. Indeed, it is reported that the vacuole, the major intracellular Ca^2+^ store, is involved in a cold-induced Ca^2+^ release^8^. In the present study, we did not calibrate the bioluminescent intensity of aequorin for [Ca^2+^]_cyt_ because of difficulties in the precise calibration, although we noted a report describing a successful calibration specific for the isoform of aequorin and temperature that the authors used^8^. It should be mentioned that although the present study has clearly suggested the involvement of MCA1 and MCA2 in cold-induced [Ca^2+^]_cyt_ increases, it remains to be examined whether complementation lines of *mca1*, *mca2* and *mca1 mca2* mutants expressing aequorin show a wild-type level of cold-induced [Ca^2+^]_cyt_ increases.

Plants employ several kinds of mechanisms to control Ca^2+^-regulated gene expression^36^. However, it is still unclear how cold-induced [Ca^2+^]_cyt_ increases are recognized. One possible mechanism involves calmodulin-binding transcription factors (CAMTAs). CAMTAs possess calmodulin (CaM)-binding domains^37^ and CAMTAs play a role in the regulation of gene expression in response to Ca^2+^ signals^38^. CAMTA3 is a positive regulator of *CBF2/DREB1C* expression and binds to the consensus sequence of a CGCG core motif, a *cis-*element for CAMTAs, in the promoter of *CBF2/DREB1C*^39^. The *camta2 camta3* double mutant is sensitive to freezing temperatures^39^. Microarray analyses demonstrated that the expression level of *HSFC1* (*At3g24520*) in the *camta1/2/3* mutant is lower than that in the wild type^40^. CAMTA is one of possibilities how increased [Ca^2+^]_cyt_ is recognized.

As shown in Fig. 1, the *mca1 mca2* double mutation reduced about 40% of the [Ca^2+^]_cyt_ increase. The transient increase in [Ca^2+^]_cyt_ may be conducted by other Ca^2+^-permeable channels that are responsible for 60% of the transient increase. One such channel could be AtGLR3.4, a member of the Arabidopsis homologs of ionotropic glutamate receptors, whose expression is up-regulated under cold stress^41^. Another such channel could be the cyclic nucleotide-gated ion channel (CNGC) family. In rice, the expressions of 10 out of 16 *CNGC* genes are induced under cold stress^42^.

The *mca1 mca2* double mutant exhibited increased sensitivity to chilling and freezing stresses, even though the single mutants did not exhibit a severe phenotype (Fig. 4). Both MCA1 and MCA2 complement a Ca^2+^ uptake deficiency of yeast cells lacking a Ca^2+^ channel composed of Mid1 and Cch1^15,16^, and generate stretch-activated currents in *Xenopus* oocytes^17.^ Even though MCA1 and MCA2 have similar functions as Ca^2+^-permeable mechanosensitive channels, their spatial expression patterns are not necessarily the same in whole plants^16^. *MCA1p::GUS* and *MCA2p::GUS* are expressed in vascular tissues of cotyledons, leaves and primary roots in common. On the other hand, *MCA1p::GUS* is expressed in the promeristem and adjacent elongation zone of the primary root, while *MCA2p::GUS* is not. *MCA2p::GUS* is expressed in mesophyll cells of cotyledons and leaves, but *MCA1p::GUS* is not. In addition, *MCA2p::GUS* is expressed more than *MCA1p::GUS* at the center of rosettes in a region corresponding to the shoot apical meristem. To survive freezing stress, the shoot apical meristem should be protected to recover plant growth^43^. Based on the observation of differences in the spatial expression patterns of *MCA1p*::*GUS* and *MCA2p*::*GUS* in whole plants, it is possible to speculate that the differences may allocate MCA1 and MCA2 a role in the acquisition of tolerance to cold stress. This allocation could explain why only the double mutant becomes hypersensitive to chilling and freezing stresses.

According to microarray analyses, several genes were up-regulated in *HSFC1*-overexpressed plants, but not in *CBF2*-overexpressed plants^30^. Some *HSFC1*-dependent and *CBF2-*independent cold-regulated genes, such as *At5g61820*, *At3g51660*, and *At4g15490*, encoding an unknown protein, a tautomerase/MIF superfamily protein, and the UDP-glycosyltransferase superfamily protein UGT84A3, respectively, were down-regulated in the *mca1 mca2* double mutant (Fig. 5). It is possible that the MCA1/2-regulated Ca^2+^ signal is transduced to a *HSFC1*-dependent pathway to enhance cold tolerance. This possibility warrants further study.

In conclusion, two mechanosensitive Ca^2+^ channels, MCA1 and MCA2, are involved in a cold-induced transient [Ca^2+^]_cyt_ increase in Arabidopsis, and in the regulation of cold tolerance through a pathway other than the *CBF/DREB1*-dependent pathway.

## Methods

### Plant materials

The Columbia-0 (Col-0) of Arabidopsis and its isogenic, transgenic lines *mca1*-null, *mca2*-null, and *mca1-*null *mca2-*null were previously described^15,16^. The complementation lines *MCA1*pro::*MCA1* in *mca1*-null *mca2*-null and *MCA2*pro::*MCA2* in *mca1*-null *mca2*-null were also previously described^15,16^.

### Monitoring of [Ca^2+^]_cyt_ changes following cold shock treatment

Apoaequorin-expressing seedlings grown at 22 °C on MS medium supplemented with 0.8% agar and 1% sucrose under 16-h light conditions at 40–60 µM m^−2^ s^−1^ light intensity were used to monitor [Ca^2+^]_cyt_ changes upon cold shock. A seedling was harvested 14 days after sowing and incubated overnight at 22 °C in 2 ml of MS medium containing 2.5 µM coelenterazine in the dark to reconstitute aequorin. The seedling was transferred to fresh MS medium (400 µl) kept at 22 °C in a tube (Microtech-Nition, #NU-063, Funabashi, Japan) and received an additional 500 µl of the same medium kept at 3, 10, or 22 °C. Luminescence (L) from aequorin in the whole seedlings was measured using a luminometer (Microtech-Nition, Model NU-2500). At the end of each monitoring, 1 ml of 20% ethanol/2 M CaCl_2_ solution was added to the medium (0.9 ml total) to measure the maximum luminescence (L_max_). The luminescence ratios (L/L_max_) are presented in Figs 1 and 2.

### Plant freezing and chilling assay

Wild-type (ecotype Col-0), *mca1*, *mca2*, and *mca1 mca2* plants were grown at 24 °C for 3 weeks in soil with fluorescent lighting (16 h/8 h light/dark photoperiod). These plants were then incubated at 4 °C for 1 week for acclimation to low temperatures. For non-acclimation, 3-week-old plants were treated with the freezing temperature without incubation at 4 °C. Whole-plant freezing assays were performed as previously described^44^. Briefly, plants were incubated at 0 °C for 1 h, and the temperature was lowered by 2 °C h^−1^ until it reached to the indicated temperature, and then held at the desired temperature for 1 h or 4 h for non-acclimated plants or cold-acclimated plants, respectively, in the incubator (IN602, Yamato Scientific Co., Ltd., Tokyo, Japan). After cold acclimation, the plants were incubated at 4 °C overnight and transferred to 24 °C. The survival ratio was determined 1 week after the freezing test.

For the chilling assay, 5-day-old plants were incubated at 4 °C. After incubation for 1 month under constant illumination, the chlorophyll content of the plants was determined. Eighty percent acetone was added to leaves ground with liquid nitrogen. The mixture was shaken at 4 °C. After centrifugation, the absorbances of the supernatant at 663 nm and 646 nm were measured. Total chlorophyll content was calculated as 17.3 *A*646 + 7.18 *A*663^45^.

Electrolyte leakage from fully developed rosettes of leaves of three-week-old plants was measured as previously described^46,47^. The sample was incubated in a refrigerated circular bath (TRL-11P, Thomas Kagaku Kikai, Co., Ltd., Japan). The conductivity was measured with a conductivity meter (CD-4302, Lutron Electronic Enterprise Co., Ltd., Taipei, Taiwan).

### RNA preparation and quantitative RT-PCR

Three-week-old wild-type (ecotype Col-0), *mca1*, *mca2*, and *mca1 mca2* plants were subjected to cold treatment at 4 °C for the indicated time. Isolation of total RNA, cDNA synthesis, and quantitative RT-PCR were performed as previously described^47^. The primers used to detect *CBF/DREB1* and its regulon genes were also previously described^47^. Other genes were detected with gene-specific primers for *At5g61820* (5′-GAGGCACCTGCGAGAAGCTTGAG-3′ and 5′ GTAACCATCTTCCCGTTTCTGTC-3′), *At3g51660* (5′-GACCTCAAAACTTAGTGATGGTG-3′ and 5′-TTAACTTGTTTGGTGATGCCTCC-3′), *At4g15490* (5′-CCTCCCATGGAAGGGACATTTGTAGA-3′ and 5′-ACAAGCAATCGCAGGATGAGCCA-3′), *MCA1* (5′-AAGATTGCCACTGCAGCATCC-3′ and 5′-ACGCCATTAGCTCATTACATGCTTC-3′), and *MCA2* (5′-AAGATCATTGCAACACCGTGGA-3′ and 5′-GTGTCTTCAAGCAAAGACAAGGTTC-3′).

## Electronic supplementary material

Supplementary Information
