# Supplementary material for: Ca2+-permeable mechanosensitive channels MCA1 and MCA2 mediate cold-induced cytosolic Ca2+ increase and cold tolerance in Arabidopsis
Source: Sci Rep. 2018 Jan 11;8:550. doi: 10.1038/s41598-017-17483-y (PMC5765038; doi:10.1038/s41598-017-17483-y)
Supplement: Supplementary file 1 — Supplementary Information [file 41598_2017_17483_MOESM1_ESM.pdf]

Supplementary Information for *Scientific Reports*

Ca^{2+}-permeable mechanosensitive channels MCA1 and MCA2 mediate cold-induced cytosolic Ca^{2+} increase and cold tolerance in Arabidopsis

Kendo Mori[1,§], Na Renhu[2,§], Maho Naito[1], Aki Nakamura[1], Hayato Shiba[2], Tsuyoshi Yamamoto[2], Takuya Suzaki[2], Hidetoshi Iida[1,*], Kenji Miura[2,*]

[2]Department of Biology, Tokyo Gakugei University, 4-1-1 Nukui kita-machi, Koganei, Tokyo 184-8501, Japan
[2]Graduate School of Life and Environmental Sciences, University of Tsukuba, Tsukuba 305-8572, Japan
[§]These authors contributed equally to this work.

[*]Correspondence to email: miura.kenji.ga@u.tsukuba.ac.jp & iida@u-gakutei.ac.jp

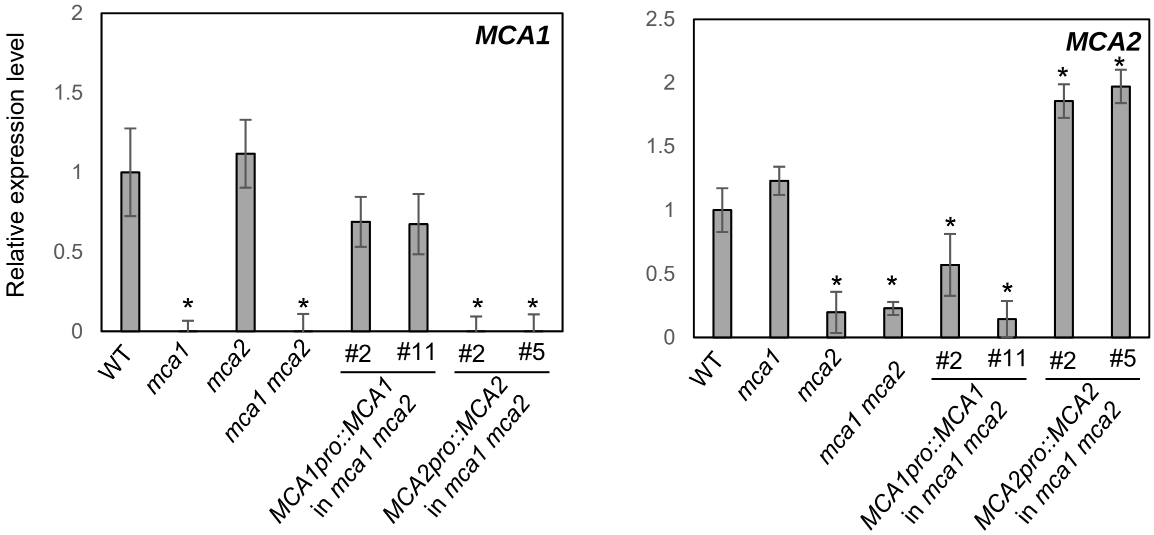

Figure S1. The levels of the *MCA1* and *MCA2* transcripts in the mutant and complement lines. Total RNA was purified from 8 plant grown for 14 days. Transcript levels of the *MCA1* and *MCA2* genes were measured by quantitative real-time PCR as described in Methods in the main text. Values represent the means ± SD (n = 3). An asterisks indicates a significant difference from wild-type plants as determined by unpaired Student's *t*-tests.
